# Crystal structure of 3-benzoyl-2-[(5-bromo-2-hydroxy-3-meth­oxy­benzyl­idene)amino]-4,5,6,7-tetra­hydro­benzo[*b*]thio­phene

**DOI:** 10.1107/S2056989015000195

**Published:** 2015-01-17

**Authors:** Manpreet Kaur, Jerry P. Jasinski, H. S. Yathirajan, Christopher Glidewell, K. Byrappa

**Affiliations:** a Department of Studies in Chemistry, University of Mysore, Manasagangotri, Mysore 570 006, India; b Department of Chemistry, Keene State College, 229 Main Street, Keene, NH 03435-2001, USA; c School of Chemistry, University of St Andrews, St Andrews, Fife KY16 9ST, Scotland; d Materials Science Center, University of Mysore, Vijyana Bhavan Building, Manasagangotri, Mysore 570 006, India

**Keywords:** crystal structure, 2-amino­thio­phene, 4,5,6,7-tetra­hydro­benzo[*b*]thio­phene, Schiff base, hydrogen bonding

## Abstract

In the cyclo­hexene ring of the title compound, C_23_H_20_BrNO_3_S, the –(CH_2_)_4_– atoms are positionally disordered [occupancy ratio = 0.753 (6):0.247 (6)]. The ring has a half-chair conformation for both the major and minor components. The dihedral angles between the mean plane of the thio­phene ring and those of the benzene and phenyl rings are 35.2 (4) and 57.7 (3)°, respectively. The planes of the two aryl rings are twisted with respect to each other by 86.4 (6)°. In the mol­ecule, there is an O—H⋯N hydrogen bond forming an *S*(6) ring motif. In the crystal, mol­ecules are linked *via* C—H⋯O hydrogen bonds, forming chains parallel to [100].

## Abstract 2

In the title compound, the planes of the benzene and phenyl rings are inclined to the thio­phene ring by 35.2 (4) and 57.7 (3)°, respectively, while the planes of the two aryl rings are almost normal to one another, making a dihedral angle of 86.4 (6)°. In the crystal, mol­ecules are linked by C—H⋯O hydrogen bonds, forming chains propagating along the *a*-axis direction.

## Chemical context   

2-Amino­thio­phene derivatives have been used in a number of applications in pesticides, dyes and pharmaceuticals. Reviews on the synthesis and properties of these compounds have been reported (Sabnis *et al.* 1999 ▸; Puterová *et al.* 2010 ▸). Schiff base compounds are an important class of compounds both synthetically and biologically. These compounds show bio­log­ical activities including anti­bacterial, anti­fungal, anti­cancer and herbicidal activities (Desai *et al.*, 2001 ▸; Karia & Parsania, 1999 ▸; Samadhiya & Halve, 2001 ▸; Singh & Dash, 1988 ▸). Furthermore, Schiff bases are utilized as starting materials in the synthesis of compounds of industrial (Aydogan *et al.*, 2001 ▸) and biological inter­est, such as β-lactams (Taggi *et al.*, 2002 ▸). The crystal and mol­ecular structures of two 2-amino­thio­phenes have been reported by our group (Kubicki *et al.*, 2012 ▸). In a continuation of our work on Schiff base derivatives of 2-amino­thio­phenes, we report herein on the synthesis and crystal structure of the title Schiff base compound.
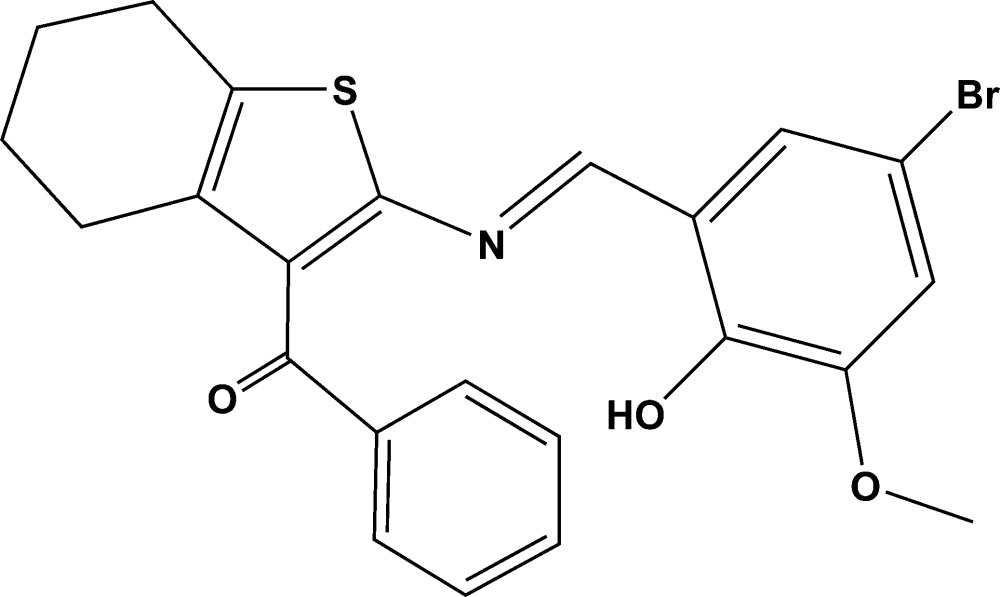


## Structural commentary   

**Figure 1:**
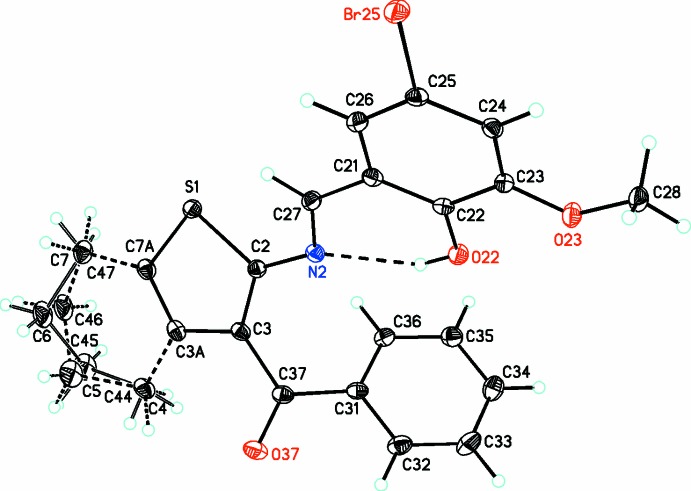
A view of the mol­ecular structure of the title compound, showing the atom labelling. Displacement ellipsoids are drawn at the 30% probability level. The intra­molecular hydrogen bond is shown as a dashed line (see Table 1 ▸ for details).

In the title compound, Fig. 1 ▸, the cyclo­hexene ring is disordered with atoms C4/C44, C5/C45, C6/C46 and C7/C47 disordered about two positions with a refined occupancy ratio of 0.753 (6):0.247 (6). Both rings (C3*A*/C4–C7/C7*A*) and (C3*A*/C44–C47/C7*A*) adopt a half-chair conformation. The mean plane of the major component (C3*A*/C4–C7/C7*A*) is slightly twisted from the mean plane of the thio­phene ring (S1/C2/C3/C3*A*/C7*A*) by 5.18 (14)°. The dihedral angles between the mean plane of the thio­phene ring and the benzene (C21–C26) and phenyl (C31–C36) rings are 35.2 (4) and 57.7 (3)°, respectively. The two aryl rings are normal to each other, making a dihedral angle of 86.4 (6)°. In the mol­ecule there is an O—H⋯N hydrogen bond forming an *S*(6) ring motif (Table 1 ▸ and Fig. 1 ▸).

## Supra­molecular features   

**Figure 2:**
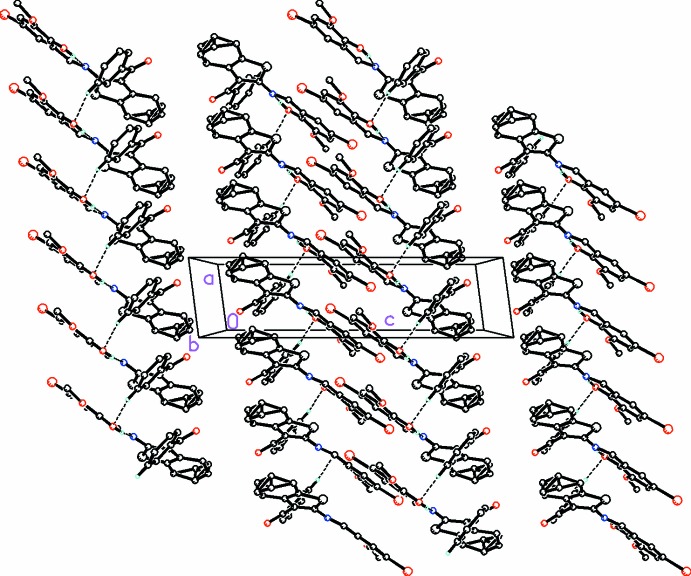
A view along the *b* axis of the crystal packing of the title compound. Dashed lines indicate weak C—H⋯O hydrogen bonds (see Table 1 ▸ for details; H atoms not involved in hydrogen bonding have been omitted for clarity).

In the crystal, mol­ecules are linked *via* C—H⋯O hydrogen bonds, observed between the benzene and phenyl rings of adjacent mol­ecules, forming chains parallel to the [100] direction (Fig. 2 ▸ and Table 1 ▸).

## Database survey   

A search of the Cambridge Structural Database (Version 5.36; Groom & Allen, 2014 ▸) for the substructure 4,5,6,7-tetra­hydro­benzo[*b*]thio­phene gave over 110 hits. Limiting the search to phen­yl(4,5,6,7-tetra­hydro­benzo[*b*]thio­phen-3-yl)methanone derivatives gave eight hits, which include five structures closely related to the title compound. These include [2-[(2-hy­droxy­benzyl­idene)amino][4,5,6,7-tetra­hydro-1-benzothio­phene-3-yl](phen­yl)methanone (I) [QOCGAS; Kaur *et al.*, 2014*a* ▸], [2-[(4-nitro­benzyl­idene)amino]-4,5,6,7-tetra­hydro-1-benzo­thio­phene-3-yl](phen­yl)methanone (II) [SODGUP; Kaur *et al.*, 2014*b* ▸], [2-(benzyl­idene­amino)-4,5,6,7-tetrahy­dro­benzo[*b*]thio­phen-3­yl](phen­yl)methanone (III) [YIYDAN; Kaur *et al.*, 2014*c* ▸], [2-[(1*H*-indol-3-yl­methylidene)amino]-4,5,6,7-tetra­hydro­benzo[*b*]thio­phen-3-yl](phen­­yl)methanone (IV) [YIWJUL; Kaur *et al.*, 2014*d* ▸] and [2-[2-bromo-5-meth­oxy­benzyl­idene)amino]-4,5,6,7-tetrahydro­benzo[*b*]thio­phene-3-yl](phen­yl)methanone (V) [CIZYIV; Kaur *et al.*, 2014*e* ▸]. Two of the compounds, (II) and (IV), crystallize in the monoclinic space group *P*2_1_, while the others, including the title compound, crystallize in centrosymmetric monoclinic space groups.

A comparison of the structural properties of the title compound to these five closely related mol­ecules reveals the following:

(*a*) The cyclo­hexene ring is disordered in compounds (II), (III), and (V), and has a slightly distorted half-chair conformation in (I), (III), (IV), and (V), and a distorted chair conformation in (II);

(*b*) The dihedral angle between the mean planes of the thio­phene and phenyl rings is 70.4 (5)° in (I), *ca*. 63.6° in (II), 65.7 (3)° in (III), 63.0 (4) and 58.8 (9)° for the two independent mol­ecules in (IV) and 66.1 (2)° in (V). The same dihedral angle in the title compound is 57.7 (3)°;

(*c*) The dihedral angle between the mean planes of the thio­phene and benzene rings is 12.1 (9)° in (I), 30.9 (8)° in (II), 8.3 (4)° in (III), 8.3 (5) and 6.7 (5)° for the two independent mol­ecules in (IV) and 9.2 (2)° in (V). In the title compound this dihedral angle is 35.2 (4)°, similar to the situation in compound (III);

(*d*) In (I), (II), (III) and (V) the benzil­idene and phenyl rings are inclined to one another by 81.0 (6), *ca*. 84.6, 73.8 (4) and 74.8 (8)°, respectively, compared to 86.4 (6)° in the title compound;

(*e*) There is an O—H⋯N intra­molecular hydrogen bond in (I), as in the title compound;

(*f*) In the crystals of (I) and (III), C—H⋯O hydrogen bonds link mol­ecules into chains along [100], as in the crystal of the title compound. In the crystal of (II), an array of C—H⋯O hydrogen bonds along [001] and [101] forms sheets parallel to (011). In the crystal of (IV), N—H⋯O hydrogen bonds link the mol­ecules, forming chains along [101]. There are also π–π stacking inter­actions present, involving the thio­phene and pyrrole rings of the two independent mol­ecules, with an inter-centroid distance of 3.468 (2) Å. In the crystal of (V), mol­ecules are linked by pairs of C—H⋯O hydrogen bonds, forming inversion dimers.

## Synthesis and crystallization   

To a solution of (2-amino-4,5,6,7-tetra­hydro-benzo[*b*]thiophen-3-yl)-phenyl­methanone (200 mg, 0.79 mmol) in 10 ml of methanol an equimolar amount of 5-bromo-2-hy­droxy-3-meth­oxy­benzaldehyde (183 mg, 0.79 mmol) was added with constant stirring. The mixture was refluxed for 6 h. A yellowish brown precipitate was obtained. Completion of the reaction was confirmed by thin layer chromatography. The precipitate obtained was filtered and dried at room temperature overnight. The solid was then recrystallized using a 1:1 solution of aceto­nitrile and di­chloro­methane, giving colourless block-like crystals.

## Refinement   

Crystal data, data collection and structure refinement details are summarized in Table 2 ▸. It was apparent from an early stage in the refinement that the saturated portion of the tetra­hydro­benzo­thio­phene unit exhibited conformational disorder over two sets of atomic sites having unequal occupancies. For the minor conformer, involving atoms C44–C47 (*cf*. Fig. 1 ▸), the bonded distances and the one-angle non-bonded distances were restrained to be the same as the corresponding distances in the major conformer, involving atoms C4–C7, subject to uncertainties of 0.005 and 0.01 Å, respectively. The atomic coordinates of atoms C4 and C44 were constrained to be identical, as were those of atoms C7 and C47. In addition, the anisotropic displacement parameters for pairs of partial-occupancy atoms occupying essentially the same physical space were constrained to be identical. The ratio of the occupancies of the disordered components refined to 0.753 (6):0.247 (6).

The H atoms in the disordered portion of the mol­ecule were included in the refinement in calculated positions, but all of the H atoms in the ordered portion of the mol­ecule were located in difference maps. All the H atoms were then treated as riding atoms in geometrically idealized positions: O—H = 0.84 Å, C—H = 0.95–0.99 Å with *U*_iso_(H) = 1.5*U*_eq_(O,C) for the hydroxyl and methyl H atoms, and = 1.2*U*_eq_(C) for other H atoms. A single weak outlier reflection (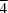,13,14) was omitted from the refinement.

## Supplementary Material

Crystal structure: contains datablock(s) I. DOI: 10.1107/S2056989015000195/su5055sup1.cif

Structure factors: contains datablock(s) I. DOI: 10.1107/S2056989015000195/su5055Isup2.hkl

Supporting information file. DOI: 10.1107/S2056989015000195/su5055Isup3.cml

CCDC reference: 1042320

Additional supporting information:  crystallographic information; 3D view; checkCIF report

## Figures and Tables

**Table 1:** Hydrogen-bond geometry (, )

*D*H*A*	*D*H	H*A*	*D* *A*	*D*H*A*
O22H22N2	0.84	2.00	2.731(3)	145
C35H35O22^i^	0.95	2.54	3.212(3)	128

Symmetry code: (i) 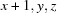.

**Table 2:** Experimental details

Crystal data	
Chemical formula	C_23_H_20_BrNO_3_S
*M*_r_	470.36
Crystal system, space group	Monoclinic, *P*2_1_/*c*
Temperature (K)	173
*a*, *b*, *c* ()	4.81267(18), 22.1919(8), 18.7012(7)
()	97.392(3)
*V* (^3^)	1980.73(13)
*Z*	4
Radiation type	Cu *K*
(mm^1^)	4.03
Crystal size (mm)	0.32 0.22 0.16

Data collection	
Diffractometer	Agilent Eos Gemini
Absorption correction	Multi-scan (*SADABS*; Sheldrick, 2008 ▸)
*T*_min_, *T*_max_	0.281, 0.525
No. of measured, independent and observed [*I* > 2(*I*)] reflections	7659, 3787, 3569
*R*_int_	0.024
(sin /)_max_ (^1^)	0.614

Refinement	
*R*[*F* ^2^ > 2(*F* ^2^)], *wR*(*F* ^2^), *S*	0.040, 0.108, 1.10
No. of reflections	3787
No. of parameters	271
No. of restraints	5
H-atom treatment	H-atom parameters constrained
_max_, _min_ (e ^3^)	0.97, 0.47

Computer programs: *CrysAlis PRO* and *CrysAlis RED* (Agilent, 2012 ▸), *SHELXS97* (Sheldrick, 2008 ▸), *PLATON* (Spek, 2009 ▸) and *SHELXL2014* (Sheldrick, 2015 ▸).

## supplementary crystallographic information

### Crystal data

**Table:**

C_23_H_20_BrNO_3_S	*F*(000) = 960
*M**_r_* = 470.36	*D*_x_ = 1.577 Mg m^−^^3^
Monoclinic, *P*2_1_/*c*	Cu *K*α radiation, λ = 1.54184 Å
*a* = 4.81267 (18) Å	Cell parameters from 3787 reflections
*b* = 22.1919 (8) Å	θ = 4.0–71.1°
*c* = 18.7012 (7) Å	µ = 4.03 mm^−^^1^
β = 97.392 (3)°	*T* = 173 K
*V* = 1980.73 (13) Å^3^	Block, colourless
*Z* = 4	0.32 × 0.22 × 0.16 mm

### Data collection

**Table:**

Agilent Eos Gemini diffractometer	3787 independent reflections
Radiation source: Enhance (Cu) X-ray Source	3569 reflections with *I* > 2σ(*I*)
Detector resolution: 16.0416 pixels mm^-1^	*R*_int_ = 0.024
ω scans	θ_max_ = 71.1°, θ_min_ = 4.0°
Absorption correction: multi-scan (*SADABS*; Sheldrick, 2008)	*h* = −5→4
*T*_min_ = 0.281, *T*_max_ = 0.525	*k* = −23→27
7659 measured reflections	*l* = −19→22

### Refinement

**Table:**

Refinement on *F*^2^	5 restraints
Least-squares matrix: full	Hydrogen site location: inferred from neighbouring sites
*R*[*F*^2^ > 2σ(*F*^2^)] = 0.040	H-atom parameters constrained
*wR*(*F*^2^) = 0.108	*w* = 1/[σ^2^(*F*_o_^2^) + (0.0688*P*)^2^ + 1.0707*P*] where *P* = (*F*_o_^2^ + 2*F*_c_^2^)/3
*S* = 1.10	(Δ/σ)_max_ = 0.001
3787 reflections	Δρ_max_ = 0.97 e Å^−^^3^
271 parameters	Δρ_min_ = −0.46 e Å^−^^3^

### Special details

**Table:**

Geometry. All e.s.d.'s (except the e.s.d. in the dihedral angle between two l.s. planes) are estimated using the full covariance matrix. The cell e.s.d.'s are taken into account individually in the estimation of e.s.d.'s in distances, angles and torsion angles; correlations between e.s.d.'s in cell parameters are only used when they are defined by crystal symmetry. An approximate (isotropic) treatment of cell e.s.d.'s is used for estimating e.s.d.'s involving l.s. planes.

### Fractional atomic coordinates and isotropic or equivalent isotropic displacement parameters (Å^2^)

**Table:**

	*x*	*y*	*z*	*U*_iso_*/*U*_eq_	Occ. (<1)
S1	0.69785 (12)	0.48921 (2)	0.30795 (3)	0.02408 (15)	
C2	0.5317 (5)	0.42375 (10)	0.27216 (12)	0.0209 (5)	
C3	0.5569 (5)	0.41833 (10)	0.19965 (12)	0.0217 (5)	
C3A	0.7019 (5)	0.46860 (11)	0.17283 (12)	0.0230 (5)	
C4	0.7525 (5)	0.47825 (12)	0.09563 (13)	0.0292 (5)	0.753 (6)
H4A	0.5712	0.4860	0.0656	0.035*	0.753 (6)
H4B	0.8335	0.4411	0.0775	0.035*	0.753 (6)
C5	0.9516 (10)	0.53138 (18)	0.0881 (2)	0.0417 (11)	0.753 (6)
H5A	1.1474	0.5171	0.0987	0.050*	0.753 (6)
H5B	0.9236	0.5457	0.0375	0.050*	0.753 (6)
C6	0.9090 (10)	0.58370 (16)	0.1374 (2)	0.0405 (10)	0.753 (6)
H6A	0.7144	0.5988	0.1267	0.049*	0.753 (6)
H6B	1.0376	0.6169	0.1287	0.049*	0.753 (6)
C7	0.9640 (6)	0.56445 (12)	0.21574 (15)	0.0316 (5)	0.753 (6)
H7A	1.1660	0.5560	0.2292	0.038*	0.753 (6)
H7B	0.9093	0.5971	0.2472	0.038*	0.753 (6)
C44	0.7525 (5)	0.47825 (12)	0.09563 (13)	0.0292 (5)	0.247 (6)
H44A	0.5808	0.4681	0.0628	0.035*	0.247 (6)
H44B	0.9051	0.4514	0.0842	0.035*	0.247 (6)
C45	0.833 (3)	0.5441 (3)	0.0844 (4)	0.0417 (11)	0.247 (6)
H45A	0.8962	0.5485	0.0364	0.050*	0.247 (6)
H45B	0.6657	0.5700	0.0856	0.050*	0.247 (6)
C46	1.063 (2)	0.5648 (5)	0.1419 (3)	0.0405 (10)	0.247 (6)
H46A	1.1222	0.6060	0.1305	0.049*	0.247 (6)
H46B	1.2268	0.5378	0.1423	0.049*	0.247 (6)
C47	0.9640 (6)	0.56445 (12)	0.21574 (15)	0.0316 (5)	0.247 (6)
H47A	1.1281	0.5659	0.2535	0.038*	0.247 (6)
H47B	0.8480	0.6006	0.2211	0.038*	0.247 (6)
C7A	0.7954 (5)	0.50878 (11)	0.22528 (13)	0.0248 (5)	
N2	0.3809 (4)	0.38630 (9)	0.31246 (10)	0.0214 (4)	
C27	0.2835 (5)	0.40820 (11)	0.36777 (12)	0.0228 (5)	
H27	0.3226	0.4492	0.3798	0.027*	
C21	0.1168 (5)	0.37392 (10)	0.41313 (12)	0.0213 (4)	
C22	0.0790 (4)	0.31190 (10)	0.40655 (11)	0.0199 (4)	
C23	−0.0965 (5)	0.28198 (10)	0.45045 (12)	0.0214 (4)	
C24	−0.2273 (5)	0.31462 (10)	0.49988 (12)	0.0216 (4)	
H24	−0.3471	0.2950	0.5291	0.026*	
C25	−0.1808 (5)	0.37664 (10)	0.50617 (12)	0.0221 (4)	
Br25	−0.34996 (6)	0.42100 (2)	0.57603 (2)	0.03072 (12)	
C26	−0.0130 (5)	0.40670 (11)	0.46400 (12)	0.0242 (5)	
H26	0.0150	0.4489	0.4691	0.029*	
O22	0.2048 (4)	0.27806 (7)	0.35966 (9)	0.0251 (3)	
H22	0.2957	0.3006	0.3350	0.038*	
O23	−0.1211 (4)	0.22165 (8)	0.44037 (10)	0.0286 (4)	
C28	−0.3190 (5)	0.19060 (11)	0.47808 (14)	0.0298 (5)	
H28A	−0.5067	0.2072	0.4638	0.045*	
H28B	−0.2674	0.1958	0.5301	0.045*	
H28C	−0.3183	0.1476	0.4661	0.045*	
C37	0.4280 (5)	0.36935 (11)	0.15198 (12)	0.0241 (5)	
O37	0.3043 (4)	0.38180 (9)	0.09281 (10)	0.0383 (5)	
C31	0.4593 (5)	0.30534 (11)	0.17603 (12)	0.0225 (4)	
C32	0.2770 (5)	0.26251 (12)	0.14146 (14)	0.0314 (5)	
H32	0.1365	0.2746	0.1038	0.038*	
C33	0.2995 (6)	0.20244 (13)	0.16173 (17)	0.0377 (6)	
H33	0.1737	0.1735	0.1384	0.045*	
C34	0.5061 (6)	0.18479 (12)	0.21618 (16)	0.0344 (6)	
H34	0.5205	0.1437	0.2302	0.041*	
C35	0.6910 (5)	0.22637 (12)	0.25018 (14)	0.0305 (5)	
H35	0.8332	0.2138	0.2872	0.037*	
C36	0.6684 (5)	0.28688 (11)	0.23010 (13)	0.0255 (5)	
H36	0.7960	0.3156	0.2533	0.031*	

### Atomic displacement parameters (Å^2^)

**Table:**

	*U*^11^	*U*^22^	*U*^33^	*U*^12^	*U*^13^	*U*^23^
S1	0.0302 (3)	0.0230 (3)	0.0203 (3)	−0.0042 (2)	0.0079 (2)	−0.0015 (2)
C2	0.0232 (11)	0.0205 (10)	0.0191 (11)	0.0011 (8)	0.0037 (8)	−0.0007 (8)
C3	0.0228 (11)	0.0244 (11)	0.0183 (11)	0.0047 (8)	0.0042 (8)	0.0013 (8)
C3A	0.0218 (11)	0.0254 (11)	0.0232 (11)	0.0045 (9)	0.0084 (8)	0.0049 (9)
C4	0.0350 (13)	0.0341 (13)	0.0204 (11)	0.0045 (10)	0.0108 (9)	0.0041 (10)
C5	0.055 (3)	0.041 (2)	0.0355 (17)	−0.005 (2)	0.030 (2)	0.0059 (15)
C6	0.056 (3)	0.0313 (19)	0.0381 (19)	−0.0044 (16)	0.0191 (19)	0.0105 (15)
C7	0.0347 (13)	0.0265 (12)	0.0356 (14)	−0.0045 (10)	0.0120 (11)	0.0022 (11)
C44	0.0350 (13)	0.0341 (13)	0.0204 (11)	0.0045 (10)	0.0108 (9)	0.0041 (10)
C45	0.055 (3)	0.041 (2)	0.0355 (17)	−0.005 (2)	0.030 (2)	0.0059 (15)
C46	0.056 (3)	0.0313 (19)	0.0381 (19)	−0.0044 (16)	0.0191 (19)	0.0105 (15)
C47	0.0347 (13)	0.0265 (12)	0.0356 (14)	−0.0045 (10)	0.0120 (11)	0.0022 (11)
C7A	0.0264 (11)	0.0251 (11)	0.0239 (11)	0.0025 (9)	0.0077 (9)	0.0034 (9)
N2	0.0231 (9)	0.0230 (9)	0.0185 (8)	−0.0013 (7)	0.0039 (7)	0.0005 (7)
C27	0.0257 (11)	0.0213 (10)	0.0222 (11)	−0.0024 (9)	0.0055 (9)	−0.0023 (9)
C21	0.0220 (10)	0.0249 (11)	0.0170 (10)	−0.0009 (9)	0.0024 (8)	0.0002 (8)
C22	0.0194 (10)	0.0249 (11)	0.0149 (9)	0.0013 (8)	0.0002 (7)	−0.0013 (8)
C23	0.0239 (11)	0.0201 (10)	0.0195 (10)	−0.0016 (8)	0.0006 (8)	0.0005 (8)
C24	0.0221 (10)	0.0249 (11)	0.0180 (10)	−0.0033 (8)	0.0036 (8)	0.0015 (8)
C25	0.0250 (11)	0.0253 (11)	0.0168 (10)	0.0004 (9)	0.0052 (8)	−0.0035 (8)
Br25	0.04036 (19)	0.02851 (18)	0.02633 (17)	−0.00364 (10)	0.01589 (12)	−0.00703 (9)
C26	0.0286 (12)	0.0232 (10)	0.0212 (10)	−0.0024 (9)	0.0044 (9)	−0.0023 (9)
O22	0.0293 (9)	0.0237 (8)	0.0240 (8)	−0.0016 (6)	0.0099 (6)	−0.0028 (6)
O23	0.0361 (9)	0.0206 (8)	0.0314 (8)	−0.0038 (7)	0.0141 (7)	−0.0009 (7)
C28	0.0344 (13)	0.0248 (11)	0.0315 (12)	−0.0091 (10)	0.0094 (10)	0.0014 (10)
C37	0.0241 (11)	0.0288 (12)	0.0198 (10)	0.0029 (9)	0.0037 (8)	−0.0023 (9)
O37	0.0496 (11)	0.0379 (10)	0.0241 (9)	0.0043 (9)	−0.0088 (8)	0.0008 (8)
C31	0.0215 (10)	0.0279 (12)	0.0185 (10)	0.0025 (9)	0.0043 (8)	−0.0048 (9)
C32	0.0270 (12)	0.0334 (13)	0.0316 (12)	0.0023 (10)	−0.0044 (10)	−0.0071 (11)
C33	0.0310 (13)	0.0318 (13)	0.0484 (16)	−0.0062 (11)	−0.0025 (11)	−0.0100 (12)
C34	0.0349 (13)	0.0248 (12)	0.0441 (15)	0.0034 (10)	0.0076 (11)	−0.0022 (11)
C35	0.0314 (12)	0.0302 (13)	0.0289 (12)	0.0060 (10)	0.0003 (10)	−0.0012 (10)
C36	0.0247 (11)	0.0257 (11)	0.0256 (11)	0.0004 (9)	0.0009 (9)	−0.0043 (9)

### Geometric parameters (Å, º)

**Table:**

S1—C7A	1.728 (2)	C21—C26	1.406 (3)
S1—C2	1.749 (2)	C22—O22	1.355 (3)
C2—C3	1.382 (3)	C22—C23	1.417 (3)
C2—N2	1.388 (3)	C23—O23	1.355 (3)
C3—C3A	1.440 (3)	C23—C24	1.387 (3)
C3—C37	1.490 (3)	C24—C25	1.397 (3)
C3A—C7A	1.359 (3)	C24—H24	0.9500
C3A—C4	1.510 (3)	C25—C26	1.372 (3)
C4—C5	1.537 (4)	C25—Br25	1.901 (2)
C4—H4A	0.9900	C26—H26	0.9500
C4—H4B	0.9900	O22—H22	0.8400
C5—C6	1.514 (5)	O23—C28	1.433 (3)
C5—H5A	0.9900	C28—H28A	0.9800
C5—H5B	0.9900	C28—H28B	0.9800
C6—C7	1.516 (4)	C28—H28C	0.9800
C6—H6A	0.9900	C37—O37	1.219 (3)
C6—H6B	0.9900	C37—C31	1.492 (3)
C7—C7A	1.501 (3)	C31—C36	1.394 (3)
C7—H7A	0.9900	C31—C32	1.395 (3)
C7—H7B	0.9900	C32—C33	1.386 (4)
C45—C46	1.512 (7)	C32—H32	0.9500
C45—H45A	0.9900	C33—C34	1.385 (4)
C45—H45B	0.9900	C33—H33	0.9500
C46—H46A	0.9900	C34—C35	1.379 (4)
C46—H46B	0.9900	C34—H34	0.9500
N2—C27	1.285 (3)	C35—C36	1.395 (4)
C27—C21	1.455 (3)	C35—H35	0.9500
C27—H27	0.9500	C36—H36	0.9500
C21—C22	1.392 (3)		
			
C7A—S1—C2	91.71 (12)	C22—C21—C26	120.5 (2)
C3—C2—N2	126.8 (2)	C22—C21—C27	122.8 (2)
C3—C2—S1	110.73 (17)	C26—C21—C27	116.7 (2)
N2—C2—S1	122.33 (17)	O22—C22—C21	122.8 (2)
C2—C3—C3A	112.5 (2)	O22—C22—C23	117.7 (2)
C2—C3—C37	124.6 (2)	C21—C22—C23	119.5 (2)
C3A—C3—C37	122.7 (2)	O23—C23—C24	124.7 (2)
C7A—C3A—C3	112.8 (2)	O23—C23—C22	115.5 (2)
C7A—C3A—C4	121.2 (2)	C24—C23—C22	119.8 (2)
C3—C3A—C4	126.0 (2)	C23—C24—C25	119.3 (2)
C3A—C4—C5	112.1 (2)	C23—C24—H24	120.4
C3A—C4—H4A	109.2	C25—C24—H24	120.4
C5—C4—H4A	109.2	C26—C25—C24	122.0 (2)
C3A—C4—H4B	109.2	C26—C25—Br25	118.62 (18)
C5—C4—H4B	109.2	C24—C25—Br25	119.34 (17)
H4A—C4—H4B	107.9	C25—C26—C21	118.9 (2)
C6—C5—C4	113.4 (3)	C25—C26—H26	120.6
C6—C5—H5A	108.9	C21—C26—H26	120.6
C4—C5—H5A	108.9	C22—O22—H22	109.5
C6—C5—H5B	108.9	C23—O23—C28	117.29 (19)
C4—C5—H5B	108.9	O23—C28—H28A	109.5
H5A—C5—H5B	107.7	O23—C28—H28B	109.5
C5—C6—C7	110.6 (3)	H28A—C28—H28B	109.5
C5—C6—H6A	109.5	O23—C28—H28C	109.5
C7—C6—H6A	109.5	H28A—C28—H28C	109.5
C5—C6—H6B	109.5	H28B—C28—H28C	109.5
C7—C6—H6B	109.5	O37—C37—C3	119.7 (2)
H6A—C6—H6B	108.1	O37—C37—C31	120.5 (2)
C7A—C7—C6	108.5 (2)	C3—C37—C31	119.8 (2)
C7A—C7—H7A	110.0	C36—C31—C32	119.2 (2)
C6—C7—H7A	110.0	C36—C31—C37	122.3 (2)
C7A—C7—H7B	110.0	C32—C31—C37	118.4 (2)
C6—C7—H7B	110.0	C33—C32—C31	120.4 (2)
H7A—C7—H7B	108.4	C33—C32—H32	119.8
C46—C45—H45A	109.3	C31—C32—H32	119.8
C46—C45—H45B	109.3	C34—C33—C32	119.8 (2)
H45A—C45—H45B	108.0	C34—C33—H33	120.1
C45—C46—H46A	109.4	C32—C33—H33	120.1
C45—C46—H46B	109.4	C35—C34—C33	120.6 (3)
H46A—C46—H46B	108.0	C35—C34—H34	119.7
C3A—C7A—C7	126.0 (2)	C33—C34—H34	119.7
C3A—C7A—S1	112.21 (18)	C34—C35—C36	119.8 (2)
C7—C7A—S1	121.76 (19)	C34—C35—H35	120.1
C27—N2—C2	118.8 (2)	C36—C35—H35	120.1
N2—C27—C21	123.9 (2)	C31—C36—C35	120.2 (2)
N2—C27—H27	118.1	C31—C36—H36	119.9
C21—C27—H27	118.1	C35—C36—H36	119.9
			
C7A—S1—C2—C3	0.59 (18)	C27—C21—C22—C23	−177.2 (2)
C7A—S1—C2—N2	−175.17 (19)	O22—C22—C23—O23	−0.3 (3)
N2—C2—C3—C3A	173.3 (2)	C21—C22—C23—O23	180.0 (2)
S1—C2—C3—C3A	−2.2 (2)	O22—C22—C23—C24	179.26 (19)
N2—C2—C3—C37	−1.4 (4)	C21—C22—C23—C24	−0.5 (3)
S1—C2—C3—C37	−176.97 (18)	O23—C23—C24—C25	178.7 (2)
C2—C3—C3A—C7A	3.3 (3)	C22—C23—C24—C25	−0.8 (3)
C37—C3—C3A—C7A	178.1 (2)	C23—C24—C25—C26	1.2 (3)
C2—C3—C3A—C4	−176.9 (2)	C23—C24—C25—Br25	−177.96 (16)
C37—C3—C3A—C4	−2.0 (4)	C24—C25—C26—C21	−0.3 (3)
C7A—C3A—C4—C5	8.5 (4)	Br25—C25—C26—C21	178.87 (17)
C3—C3A—C4—C5	−171.4 (3)	C22—C21—C26—C25	−1.0 (3)
C3A—C4—C5—C6	−37.5 (4)	C27—C21—C26—C25	177.6 (2)
C4—C5—C6—C7	61.4 (5)	C24—C23—O23—C28	6.8 (3)
C5—C6—C7—C7A	−51.5 (4)	C22—C23—O23—C28	−173.6 (2)
C3—C3A—C7A—C7	177.3 (2)	C2—C3—C37—O37	133.9 (3)
C4—C3A—C7A—C7	−2.6 (4)	C3A—C3—C37—O37	−40.3 (3)
C3—C3A—C7A—S1	−2.8 (3)	C2—C3—C37—C31	−48.1 (3)
C4—C3A—C7A—S1	177.36 (18)	C3A—C3—C37—C31	137.7 (2)
C6—C7—C7A—C3A	24.3 (4)	O37—C37—C31—C36	158.4 (2)
C6—C7—C7A—S1	−155.6 (2)	C3—C37—C31—C36	−19.6 (3)
C2—S1—C7A—C3A	1.30 (19)	O37—C37—C31—C32	−19.7 (4)
C2—S1—C7A—C7	−178.8 (2)	C3—C37—C31—C32	162.3 (2)
C3—C2—N2—C27	−152.3 (2)	C36—C31—C32—C33	1.4 (4)
S1—C2—N2—C27	22.7 (3)	C37—C31—C32—C33	179.6 (2)
C2—N2—C27—C21	178.5 (2)	C31—C32—C33—C34	−0.6 (4)
N2—C27—C21—C22	9.0 (4)	C32—C33—C34—C35	−0.4 (5)
N2—C27—C21—C26	−169.6 (2)	C33—C34—C35—C36	0.6 (4)
C26—C21—C22—O22	−178.3 (2)	C32—C31—C36—C35	−1.2 (4)
C27—C21—C22—O22	3.1 (3)	C37—C31—C36—C35	−179.3 (2)
C26—C21—C22—C23	1.4 (3)	C34—C35—C36—C31	0.2 (4)

### Hydrogen-bond geometry (Å, º)

**Table:**

*D*—H···*A*	*D*—H	H···*A*	*D*···*A*	*D*—H···*A*
O22—H22···N2	0.84	2.00	2.731 (3)	145
C35—H35···O22^i^	0.95	2.54	3.212 (3)	128

Symmetry code: (i) *x*+1, *y*, *z*.

## References

[bb1] Agilent (2012). *CrysAlis PRO* and *CrysAlis RED*. Agilent Technologies, Yarnton, England.

[bb2] Aydogan, F., Ocal, N., Turgut, Z. & Yolacan, C. (2001). *Bull. Korean Chem. Soc.* **22**, 476–480.

[bb3] Desai, S. B., Desai, P. B. & Desai, K. R. (2001). *Heterocycl. Commun.* **7**, 83–90.

[bb4] Groom, C. R. & Allen, F. H. (2014). *Angew. Chem. Int. Ed.* **53**, 662–671. 10.1002/anie.201306438 24382699

[bb5] Karia, F. D. & Parsania, P. H. (1999). *Asian J. Chem.* **11**, 991–995.

[bb6] Kaur, M., Jasinski, J. P., Kavitha, C. N., Yathirajan, H. S. & Byrappa, K. (2014*a*). *Acta Cryst.* E**70**, o476–o477. 10.1107/S1600536814006199 PMC3998550 24826172

[bb7] Kaur, M., Jasinski, J. P., Kavitha, C. N., Yathirajan, H. S. & Byrappa, K. (2014*b*). *Acta Cryst.* E**70**, o738–o739. 10.1107/S1600536814012185 PMC4051066 24940302

[bb8] Kaur, M., Jasinski, J. P., Kavitha, C. N., Yathirajan, H. S. & Byrappa, K. (2014*c*). *Acta Cryst.* E**70**, o507–o508. 10.1107/S1600536814006679 PMC4011224 24860324

[bb9] Kaur, M., Jasinski, J. P., Yamuna, T. S., Yathirajan, H. S. & Byrappa, K. (2014*d*). *Acta Cryst.* E**70**, o501–o502. 10.1107/S1600536814006345 PMC3998588 24826189

[bb10] Kaur, M., Jasinski, J. P., Yamuna, T. S., Yathirajan, H. S. & Byrappa, K. (2014*e*). *Acta Cryst.* E**70**, o581–o582. 10.1107/S1600536814008290 PMC4011278 24860382

[bb11] Kubicki, M., Dutkiewicz, G., Yathirajan, H. S., Dawar, P., Ramesha, A. R. & Dayananda, A. S. (2012). *Crystals*, **2**, 1058–1066.

[bb12] Puterová, Z., Krutošiková, A. & Végh, D. (2010). *Arkivoc*, **(i)**, 209–246.

[bb13] Sabnis, R. W., Rangnekar, D. W. & Sonawane, N. D. (1999). *J. Heterocycl. Chem.* **36**, 333–345.

[bb14] Samadhiya, S. & Halve, A. (2001). *Orient. J. Chem.* **17** 119–122.

[bb15] Sheldrick, G. M. (2008). *Acta Cryst.* A**64**, 112–122. 10.1107/S0108767307043930 18156677

[bb16] Sheldrick, G. M. (2015). *Acta Cryst.* C**71**, 3–8.

[bb17] Singh, W. M. & Dash, B. C. (1988). *Pesticides*, **22**, 33–37.

[bb18] Spek, A. L. (2009). *Acta Cryst.* D**65**, 148–155. 10.1107/S090744490804362X PMC2631630 19171970

[bb19] Taggi, A. E., Hafez, A. M., Wack, H., Young, B., Ferraris, D. & Lectka, T. (2002). *J. Am. Chem. Soc.* **124**, 6626–6635. 10.1021/ja0258226 12047183

